# Trehalose prevents aggregation of exosomes and cryodamage

**DOI:** 10.1038/srep36162

**Published:** 2016-11-08

**Authors:** Steffi Bosch, Laurence de Beaurepaire, Marie Allard, Mathilde Mosser, Claire Heichette, Denis Chrétien, Dominique Jegou, Jean-Marie Bach

**Affiliations:** 1 IECM, EA4644 Nantes University, ONIRIS, USC1383 INRA, Nantes, France; 2 IGDR, UMR6290 CNRS, University of Rennes 1, Rennes, France; 3 MRIC-Biosit, UMS3480 CNRS, University of Rennes 1, Rennes, France

## Abstract

Exosomes are important mediators in intercellular communication. Released by many cell types, they transport proteins, lipids, and nucleic acids to distant recipient cells and contribute to important physiopathological processes. Standard current exosome isolation methods based on differential centrifugation protocols tend to induce aggregation of particles in highly concentrated suspensions and freezing of exosomes can induce damage and inconsistent biological activity. Trehalose is a natural, non-toxic sugar widely used as a protein stabilizer and cryoprotectant by the food and drug industry. Here we report that addition of 25 mM trehalose to pancreatic beta-cell exosome-like vesicle isolation and storage buffer narrows the particle size distribution and increases the number of individual particles per microgram of protein. Repeated freeze-thaw cycles induce an increase in particle concentration and in the width of the size distribution for exosome-like vesicles stored in PBS, but not in PBS 25 mM trehalose. No signs of lysis or incomplete vesicles were observed by cryo-electron tomography in PBS and trehalose samples. In macrophage immune assays, beta-cell extracellular vesicles in trehalose show consistently higher TNF-alpha cytokine secretion stimulation indexes suggesting improved preservation of biological activity. The addition of trehalose might be an attractive means to standardize experiments in the field of exosome research and downstream applications.

Exosomes are naturally occurring membrane vesicles formed by inward budding within late endosomes and released into the extracellular space upon fusion of the endosome to the plasma membrane^1^,^2^. About 30 to 100 nm in size, they contain proteins, lipids and nucleic acids derived from their cell of origin. Since their discovery in 1983, compelling evidence has supported the importance of exosomes in intercellular communication in a broad range of physiological and pathological settings^3^,^4^,^5^ (reviewed in ref. 6). In the immune system, uptake of exosomes by antigen-presenting cells induces immunogenic or tolerogenic responses. This way, exosomes released by pancreatic insulin-secreting beta-cells promote inflammation and further autoimmune diabetes through transfer of auto-antigens or immune stimulatory microRNAs sequences^7^. Exosomes hold numerous potential applications in diagnostics and therapeutics, prompting interest in exosome research to explore hitherto uncharted characteristics.

Obviously, robust exosome isolation procedures are the cornerstone on which meaningful results are built. Common methods of purification employ ultracentrifugation, filtration, polymeric precipitation, size exclusion, and immune-isolation- and microfluidic techniques. Differential centrifugation-based protocols in combination with sucrose cushions or gradients are the standard choice, because they warrant highly pure vesicle preparations and avoid the introduction of exogenous contaminants like chemical precipitation reagents^8^,^9^,^10^. However, extracellular vesicles suffer from aggregation and flocculation when stored in physiological saline solutions and degrade during freezing/thawing. It is currently advisable to proceed to biological assays immediately after collection, a restraining factor in large-scale or clinical trials.

Trehalose is a natural, non-reducing disaccharide sugar contained in mushrooms, shrimps, insects and bacteria. Trehalose is widely used as a texturizer, stabilizer or humectant by the food and cosmetic industry^11^ and as a (cryo-) preservative for labile protein drugs, vaccines and liposomes as well as for cells and organs for transplantation^12^,^13^,^14^,^15^,^16^. Multiple toxicity studies established the safety and tolerance of trehalose in mice and humans after oral, gastric or parenteral administration (International Programme on Chemical Safety www.inchem.org/documents/jecfa/jecmono/v46je05.htm)^17^,^18^. Interesting bioprotective actions offered by trehalose include its ability to stabilize proteins, cell membranes and liposomes, to decrease intracellular ice formation during freezing, and to prevent protein aggregation (reviewed in ref. 11). Animal models show the therapeutic benefit of trehalose in inhibiting protein aggregation in Huntington’s, Alzheimer’s, and prion protein disease^11^. Despite having a glycaemic index similar to glucose, trehalose mitigates insulin secretion in humans and insulin resistance, metabolic syndromes and developmental defects linked to maternal diabetes in mice^19^,^20^,^21^,^22^. In the field of exosomes, trehalose has been used successfully to minimize fusion in *in vitro* electroporation experiments^23^ and loss of exosomes during freeze-drying (patent CN104488850A). There is a general consensus that trehalose physically shields proteins and cells, although different theories of the underlying mechanism through vitrification, preferential exclusion or water replacement have been proposed^11^.

This study attempts to evaluate the potential benefits of 25 mM trehalose in PBS (TRE) to maintain dispersal, functionality and stability of beta-cell exosome-like vesicles (beta-ELV) during isolation and storage. In comparison to PBS alone, the addition of trehalose reduces aggregation of ELVs, and preserves integrity during freezing and thawing. For these reasons, trehalose might help to improve the integrity and stability of extracellular vesicles required in standardized large-scale applications.

## Results

### Characterization of ELVs released by pancreatic beta-cells

**Figure 1:**
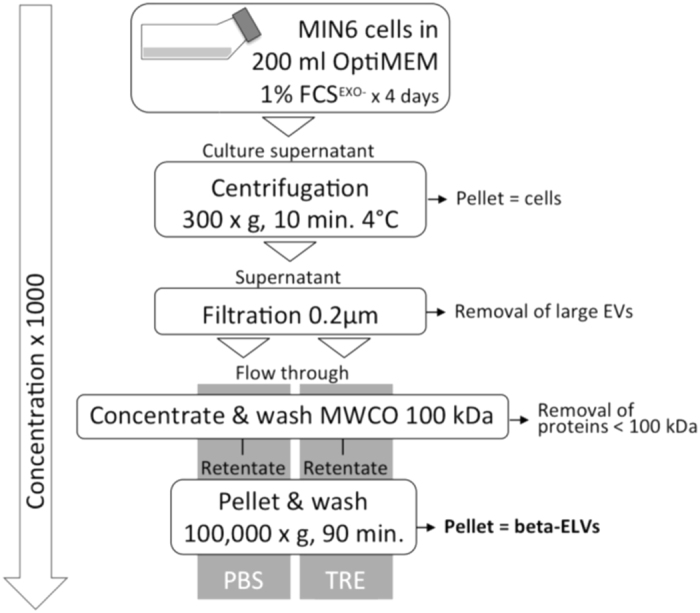
Purification of beta-ELVs using differential centrifugation and ultrafiltration. Four-day supernatants from MIN6 beta-cell cultures grown in OptiMEM 1% FCS exosome-depleted media were centrifuged and filtered through a 0.2 μm filter sterilization device to remove cells and large debris. Next, supernatants were split and concentrated on an ultracel-membrane with a molecular weight cut off (MWCO) of 100 kDa and washed three times with 15 ml of either PBS or TRE. Approximately 300 μl of concentrate were recovered, re-suspended in 10 ml of wash buffer and pelleted by ultracentrifugation twice. The final ELV-pellet was recovered in approximately 1 μl of PBS or TRE per ml of culture supernatant. ELVs were stored at 4 °C for one day or at −80 °C for up to one year. ELVs were routinely assessed for particle size distribution, protein and RNA content, and biological activity.

ELVs released by murine insulinoma MIN6 beta-cells^24^ were isolated using differential centrifugation and ultrafiltration steps outlined in Fig. 1.

**Figure 2:**
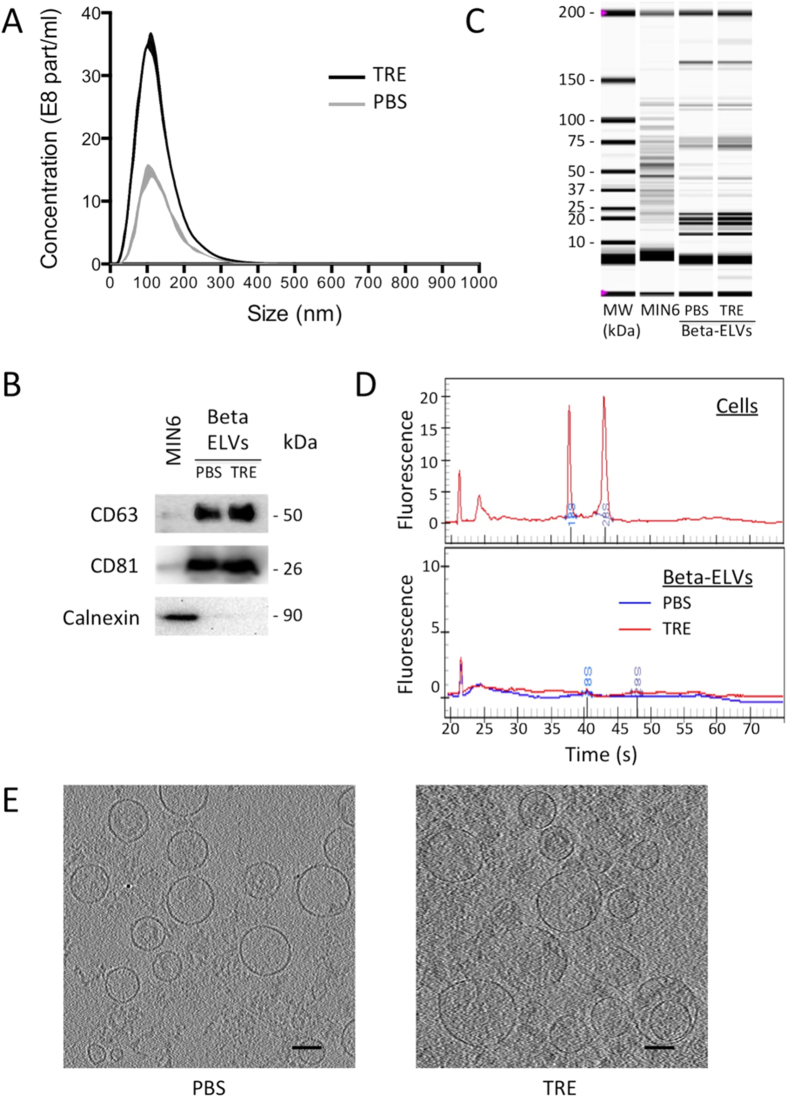
TRE does not alter MIN6 insulinoma cell-derived exosome characteristics. (**A**) Particle size distribution of beta-ELVs measured by nanoparticle tracking analysis. The size distribution curve fits average particle size and concentration. Data from one representative sample out of twelve are shown. (**B**) Western blot analysis of 15 μg of total cell lysates (MIN6) or beta-ELV proteins reveal the presence of canonical exosomal protein markers and the absence of cellular calnexin. (**C**,**D**) Experion automated electrophoresis analysis of (**C**) protein profiles under reducing conditions, and (**D**) total RNA isolated from MIN6 cells (upper panel) and of beta-ELVs in PBS or TRE (lower panel). (**E**) Cryo-electronic tomography images of fully hydrated, unstained beta-ELV isolated in PBS or TRE show a population of membrane vesicles of heterogeneous shape and sizes. Scale bars: 100 nm.

Nanoparticle tracking analysis (NTA) of beta-ELVs showed a positively skewed bell-shaped particle size distribution (PSD) peaking at mode 113 nm (46–130) or 111 nm (102–122) (median and (range)) for PBS or TRE, respectively (Fig. 2A, Supplementary Table S1, and Supplementary Fig. S1). As NTA measures the hydrodynamic diameter from the diffusional properties of particles in solution, the observed size is consistent with the theoretical size of exosomes and observations made by others^25^,^26^,^27^. Western blot analysis revealed the presence of canonical exosomal markers, i.e. tetraspanins CD63 and CD81 enriched in beta-ELVs in comparison to MIN6 whole cell lysates (Fig. 2B). Similar results were obtained for beta-ELVs isolated using PBS or TRE. To measure the amount of co-isolated non-vesicular material, the endoplasmic reticulum protein calnexin was assessed. Its absence from beta-ELVs indicates production with a low degree of contamination by cellular and microsomal components. Application of beta-ELVs to an Optiprep density ultracentrifugation gradient followed by western blot analysis led to the detection of a strong signal for the tetraspanin CD81 in fractions with a density of 1.16 g/ml for both PBS-ELVs and TRE-ELVs (Supplementary Fig. S2). Qualitative and quantitative differences in protein patterns could be distinguished between beta-ELVs and whole cell lysates of the parental MIN6 cell line on electropherograms obtained under reducing conditions (Fig. 2C). These nanovesicles enclosed mainly small RNA molecules, whereas ribosomal RNAs, predominant in MIN6 cells, nearly completely disappeared (Fig. 2D). Cryo-electron tomography allows the reconstruction of undistorted three-dimensional images of biological samples trapped in non-crystalline ice in the absence of chemical fixation and dehydration that might damage fragile vesicles. Tomography images of freshly isolated beta-ELVs (Fig. 2E and Supplementary Fig. S3) illustrate the existence of numerous almost circular nanovesicles, clearly delimited by a phospholipid membrane similarly for PBS and TRE samples. Individual vesicles show physical heterogeneity with respect to morphology, size, cargo, and surface proteins. The examination of 329 individual particles per sample on cryo-electron microscope tomograms revealed 84% or 87% of single circular vesicles with an average size of 55 nm (26–205) or 68 nm (29–216) (median and (range)), for PBS and TRE, respectively. No signs of lysis, i.e. incomplete vesicles, were observed. Oval shaped vesicles accounted for up to 5% of all vesicles. Occasionally, small aggregates composed of a few vesicles were detected. One important finding is the presence of larger vesicular structures (≤2%) composed of interlocked smaller vesicles suggesting that fusion might yield vesicles in the micrometric range. Rare, non ELV-like material included virus-like particles presenting a dense circular core and regularly spaced, protruding surface proteins, smaller elements resembling clathrin-triskelion structures and non-vesicular amorphous material.

The size, buoyant density, protein and RNA content of beta-ELVs are characteristic of exosomes and collectively suggest that ELVs were the main product obtained using our isolation protocol^8^.

### Effects of TRE on beta-ELVs colloidal stability

**Figure 3:**
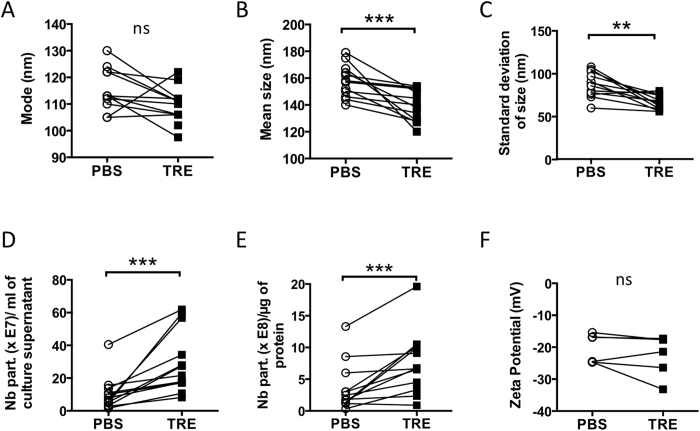
TRE reduces aggregation of beta-ELVs. (**A**–**E**) NTA of beta-ELVs isolated using either PBS or TRE buffer; n = 12 independent samples; Wilcoxon two-tailed matched pairs test. (**A**) While the particle mode remained unchanged (p = 0.0566), addition of trehalose significantly decreased (**B**) mean particle size (***p < 0.001) and (**C**) standard deviation (**p < 0.01). This reduction in size paralleled an increase in (**D**) particle recovery and (**E**) the number of particles per μg of protein obtained. (**F**) Zeta potential was measured on a qNano instrument (Izon) on n = 6 independent samples; Wilcoxon two-tailed matched pairs test, ns (p = 0.3125).

To track the effects of TRE on the stability of ELV-suspensions, the apparent particle size, number and zeta potential were measured for beta-ELVs isolated from MIN6 culture supernatants using either PBS or TRE (Fig. 3, Supplementary Table S1 and Supplementary Fig. S1).

The peak of the frequency distribution (mode) remained unchanged for beta-ELVs isolated and stored in either PBS or TRE buffer. However, the use of TRE caused a sharp drop in the mean particle size (p = 0.0005) and narrowed the spread of the size distribution as shown by the lowered standard deviation (p = 0.002) and span (p = 0.023). The median particle size (D50) was reduced by 13 nm (p = 0.0015), i.e. approximately one-tenth of the modal size. Preliminary NTA results on serum-derived vesicles suggest that trehalose may also reduce the size and standard deviation of ELVs of different origins (data not shown).

**Figure 4:**
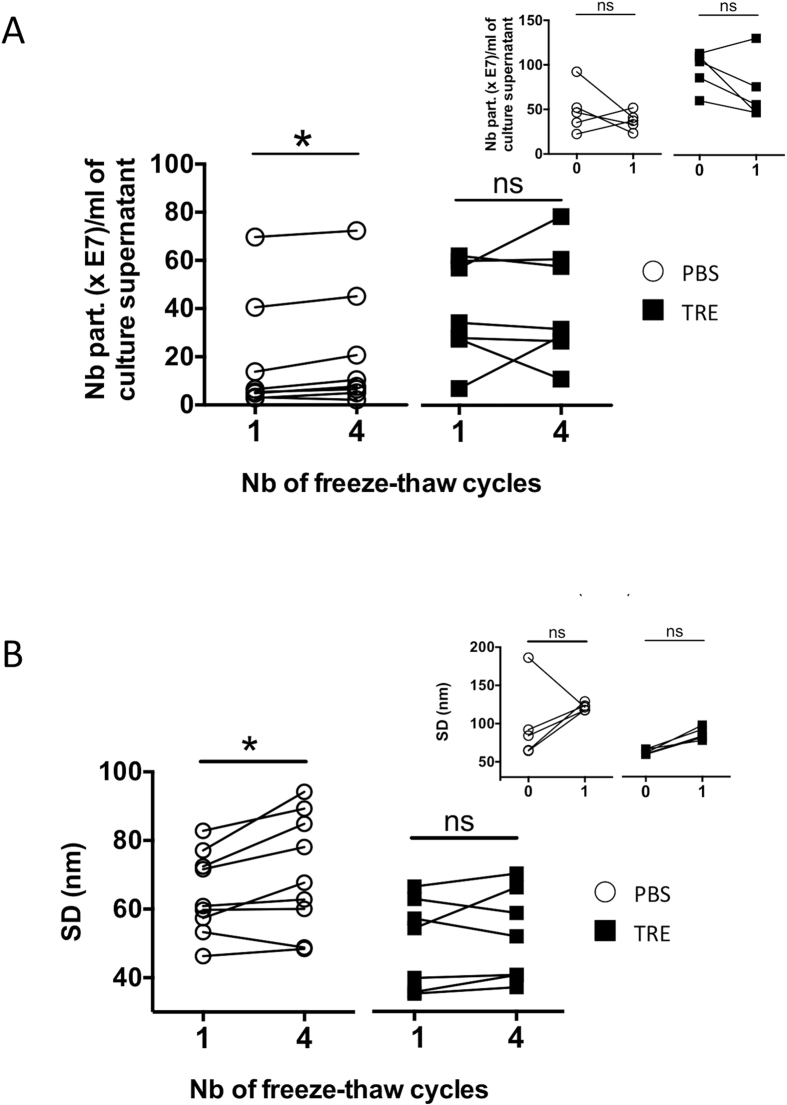
TRE protects against alteration of particle structure induced by freezing. (**A**,**B**) NTAv3.1 analysis of fresh versus freeze-thawed beta-ELVs. Main graphs depict results obtained for beta-ELVs after one or four freeze-thaw cycles (n = 7–8 biological replicates; Wilcoxon two-tailed matched pairs test). The PSD’s standard deviation and particle number recovered per ml of culture supernatant increased significantly for PBS ELVs, but remained unchanged for TRE ELVs (*p < 0.05). Graph insets show results obtained for fresh beta-ELVs or after one freeze-thaw cycle in an independent experiment (n = 5 biological replicates; Wilcoxon two-tailed matched pairs test showed no significant differences).

Concomitantly, TRE samples exhibited higher particle concentrations and yields (Fig. 3D,E). The amount of particles produced per ml of culture supernatant was three-fold higher using TRE compared to PBS (p = 0.0005). On average, 0.4 μg (0.1–0.7) or 0.4 μg (0.2–1.0) of protein (median and (range)) were recovered per 10^6^ MIN6 cells for ELVs in PBS or TRE, respectively. These yields are slightly lower than those obtained by differential centrifugation by Sheng *et al*. for MIN6 cells^28^, but in line with common estimates of 0.05–0.5 μg per 10^6^ cells for various cell lines^8^. The purity of vesicle preparations is commonly expressed as the ratio of vesicle count to relative amount of protein. The use of TRE led to a three-fold increase in the number of particles (median) produced per μg of protein (p = 0.001) (Fig. 4E). Together, cryo-electron tomography, the reduced positive skew of the PSD curve and the increased particle number of TRE-ELVs suggest that TRE may limit particle aggregation and fusion.

Next, beta-ELVs were subjected to tunable resistive pulse sensing of individual particle surface charges (zeta potential) on a qNano instrument (Izon, Fig. 3F). When the absolute value of the charge at the surface of a particle in a liquid approaches zero, electrostatic repulsion between particles fails to counteract naturally occurring attractive forces leading to aggregation. In general, a zeta potential with an absolute value greater than 20–30 mV warrants colloidal stability. Weakly negative zeta potentials of −20.7 mV (−24.7–(−15.4)) and −19.6 mV (−33.2–(−17.3)) (median, range) were recorded for beta-ELVs in PBS or TRE, respectively.

### Comparison of beta-ELVs integrity after freezing and thawing in PBS or TRE

ELV integrity can be disrupted by repeated freeze/thaw cycles^29^. The use of fresh exosomes remains the best choice for functional assays, but sample acquisition and time-consuming procedures for ELV isolation might be practical concerns. Trehalose is a well-known cryoprotectant. To evaluate the impact of TRE on beta-ELV integrity and stability, NTA was performed before and after one or four cycles of freezing and thawing (Fig. 4). No measurable differences in the PSD and concentration were observed after the first freeze-thaw step. However, repeating freezing and thawing four times induced a slight increase in the standard deviation of the PSD (p = 0.0273) and particle number (p = 0.0391) for beta-ELVs stored in PBS in contrast to beta-ELVs stored in TRE.

### Immune-stimulatory activity of PBS and TRE beta-ELVs

**Figure 5:**
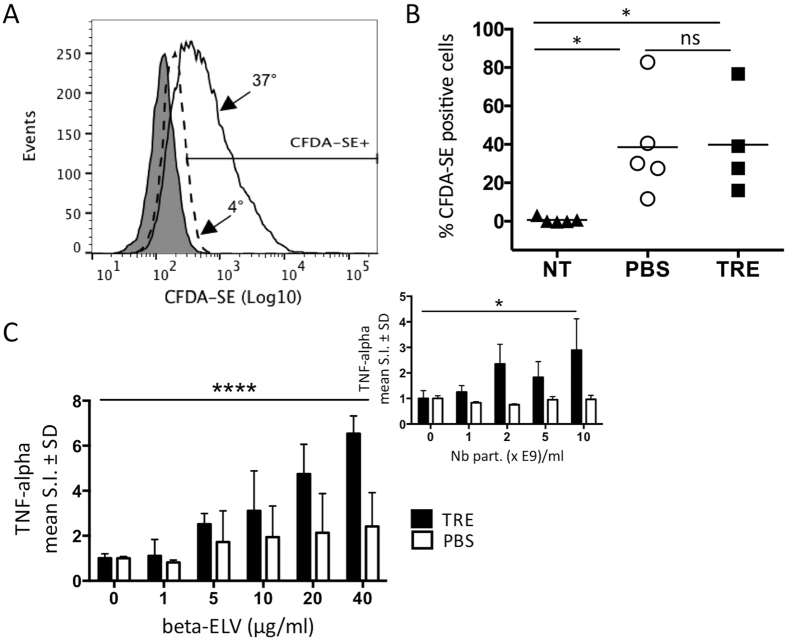
Beta-ELV uptake and immune-stimulatory activity. (**A**,**B**) RAW264.7 macrophage suspension cultures were incubated for six hours in the presence of 20 μg/ml of freshly isolated CFDA-SE labelled beta-ELVs at 37 °C or 4 °C. Internalisation was monitored by flow cytometry analysis gating on 7-AAD negative events. (**A**) Clear histograms represent CFDA-SE fluorescence intensities for beta-ELVs (PBS) treated cells at 37 °C (solid line) or 4 °C (dashed line). Grey shading indicates auto-fluorescence of untreated cells. Data for one representative experiment out of five are shown. (**B**) Comparison of CFDA-SE positive population between beta-ELV treated cells and no treatment controls depicted as % CFDA-SE^+^ cells at 37 °C - % CFDA-SE^+^ cells at 4 °C (n = 4–5 from five independent experiments). Kruskal-Wallis test * p < 0.05, ns (p > 0.9999). (**C**) TNF-alpha cytokine secretion in culture supernatants of RAW264.7 macrophages stimulated with defrosted beta-ELVs for 18 h as measured by ELISA. Results are represented as mean TNF-alpha stimulation indexes (S.I.) of biological replicates ± SD (n = 7–8). Stimulation index was calculated as the difference between TNF-alpha levels measured in exosome-treated versus untreated macrophage cultures. Significant differences between PBS and TRE exosomes stimulation indexes were calculated using two-way ANOVA (****p < 0.0001 and *p = 0.0401 for doses of ELVs in μg/ml or particle count/ml, respectively). Data from one (inset) or two (main graph) representative experiments are shown.

Taking advantage of MIN6 ELVs’ ability to steer immune responses^7^,^28^,^30^, we used murine RAW264.7 macrophages to assess for biological activity. When treated with 20 μg/ml of fresh fluorescently labelled beta-ELVs for six hours and analysed by flow cytometry, beta-ELV uptake led to an increase in CFDA-SE positive RAW264.7 cells in 43.0% of beta-ELV treated cells at 37 °C (Fig. 5A). Beta-ELV treated controls grown at 4 °C presented only 0.5% CFDA-SE-positive cells. The median fluorescence intensity increased more than 7-fold in beta-ELV -treated cells at 37 °C compared to treated cells at 4 °C suggesting an energy-dependent mechanism of internalisation rather than passive adsorption. TRE did not alter the kinetics of beta-ELV uptake at this working concentration as shown by similar percentages of CFDA-SE positive cells in five independent experiments (Fig. 5B).

Next, we compared the aptitude to trigger cytokine release of beta-ELVs after prolonged storage at −80 °C in PBS or TRE. Briefly, RAW264.7 macrophages were treated with beta-ELVs at concentrations ranging from 1 to 40 μg/ml. For TRE beta-ELVs, final concentration of trehalose in culture medium was kept constant at 2mM in all situations with no effect on cell viability (data not shown). As depicted in Fig. 5C, beta-ELVs induced TNF-alpha cytokine release from RAW264.7 macrophages (p < 0.0001). The influence of TRE on exosome-related TNF-alpha secretion was analysed by a two-way ANOVA test including isolation/storage buffer group, exosome concentration, and the interaction between these two parameters. TRE-ELVs had significantly higher TNF-alpha stimulation indexes compared to PBS-ELVs (p < 0.0001 and p = 0.0401 for dose of ELV in μg/ml or particle count/ml, respectively) with 14% of total variation due to the interaction between ELV dose and buffer group (two-way ANOVA, p = 0.0007 and p = 0.0401 for dose of ELVs in μg/ml and particle count/ml, respectively).

## Discussion

Approaches to preserve physical characteristics of extracellular vesicles in solutions are crucial to warrant biological activity and reproducibility in downstream applications. Recurrent problems are linked to the inherent risk of particle aggregation during high-speed centrifugation in standard isolation procedures and storage of highly enriched extracellular vesicle suspensions promoting their interaction, as well as to damage caused by freezing^8^,^25^,^31^,^32^. Consequently, the overall particle yield provides limited information on the quantity of biological active material available. Here we evaluate the aptitude of trehalose, a natural, non-toxic dispersion agent and cryoprotectant to preserve stability of ELV suspensions using protein, PSD, cryo-electron tomography, zeta potential, and cytokine secretion analyses. TRE (25 mM) is simply added to extracellular vesicle isolation and storage buffer with no notable effect on the length and cost of the procedure. The impact on beta-ELV stability is assessed in a standard ultracentrifugation-based isolation protocol achieving a final concentration factor of 1,000. TRE and PBS beta-ELVs present similar protein and RNA profiles and typical exosomal markers, and are free of cellular calnexin contaminants. Like exosomes, both PBS- and TRE-ELVs migrate at a buoyant density of 1.16 g/ml in iodixanol density gradients. Cryo-electron tomograms of freshly isolated beta-ELVs in both PBS and TRE reveal heterogeneous pleiomorphic populations of mainly circular nanovesicles clearly delimitated by a bilayered lipid membrane. The median size of single vesicles was 55 nm or 68 nm for PBS- or TRE-ELVs. In line with NTA results, larger multi-vesicular structures composed of interlocked vesicles were present on most sections. In contrast, aggregates of independent vesicles were rarely observed suggesting that fusion may be the prevalent mechanism of formation of larger particles. Oval vesicles were observed in areas of high vesicular density raising the question of whether spatial constraints may affect the vesicular shape. The presence of extra-vesicular structures like clathrin triskelions and amorphous material indicates co-purification of material derived from the endosome, other cellular (intra-) vesicular compartments or the plasma membrane. A few virus-like particles presenting regularly spaced surface proteins and/or a central core were detected on several tomograms in PBS and TRE samples. Although MIN6 cells do not produce infectious virions, they express viral sequences^33^. Further purification of defined sub-populations with distinct biological properties based on floating behaviour^34^ or immune-caputure^8^ shall permit to identify the precise nature of these entities. The considerable diversity of vesicles is reminiscent of previous observations made for extracellular vesicles isolated from cell culture supernatant^34^,^35^ and biological fluids^36^,^37^.

Compared to PBS, TRE generates a significantly higher particle count with reduced mean size and spread as shown by NTA. The purity of extracellular vesicle preparations is commonly calculated by the ratio of its particle to protein concentration^38^. According to this ratio, the median purity of ELVs isolated and stored in TRE is three times higher than for ELVs in PBS. NTA is based on scattering a laser beam by particles in suspension. A CCD camera mounted on a microscope records the mean velocity of each particle, which allows calculation of the overall particle size that is inversely correlated to Brownian motion by the Stokes-Einstein equation. Therefore, NTA analysis does not discriminate between a single large particle and aggregates or fusion products of several smaller particles. For spherical particles, an n-fold increase in the diameter leads to an n^3^-fold increase in the volume. Consequently, the presence of multi-vesicular structures like those detected by cryo-electron tomography in our samples, weighs heavily on particle count accuracy. Asymmetric distributions point to aggregation and materials with narrower distributions are less prone to segregation. We obtained a Pearson’s skew of 1.5 (1.1–4.3) and 1.3 (0.9–1.8) and a span of 1.4 (1.1–1.9) and 1.2 (1.0–1.4) for PBS and TRE beta-ELVs (median (range)), respectively. Although we cannot rule out an improved performance of ultrafiltration and particle recovery in the presence of trehalose, the reduced positive skew and span of the PSD curve support the idea of improved particle separation in the presence of TRE. According to our investigations, TRE, a non-reducing sugar, does not impact upon the absolute particle surface charge, i.e. its zeta potential that typically correlates with suspension stability. Zeta potentials measured for beta-ELVs were weakly negative in line with recordings made by others on extracellular vesicles in PBS^39^,^40^. Probably, trehalose adds stringency by shielding attractive forces between particles in a high salt concentration environment and maintaining enough repulsion to keep the particles apart. In plants and bacteria, trehalose has been reported to confer tolerance to high salt stress^41^,^42^,^43^.

Different studies have produced conflicting results regarding the resistance of extracellular vesicles to freezing. NTA and electron microscopy analysis of exosomes after one to ten freeze and thaw cycles at −80 °C showed no change in vesicle size^44^. On the contrary, losses of 10 to 15% of plasma extracellular vesicle were observed after a single freeze and thaw step using flow cytometry analysis^45^. Our NTA results show a slight, significant increase in the number of particles and in the standard deviation of the size distribution for beta-ELVs in PBS after repeated freeze and thaw cycles (Fig. 4). No changes were visible after a single round of freezing or were below the detection limits of this technique. Freezing may also induce membrane damage and leakage of vesicular content in the absence of perceivable changes of size and concentration. Loss of function following freezing remains a matter of debate in the field of extracellular vesicle research^10^. Earlier, we showed that freshly isolated beta-ELVs in PBS induce dose-dependent cytokine release from murine RAW264.7 macrophages^7^. Here, we compare the ability of beta-ELVs to stimulate TNF-alpha secretion after storage at −80 °C for up to 1 month in PBS or TRE. Evidence exists that trehalose at concentrations of 5–7.5% (w/v) either does not affect immune function or exerts anti-inflammatory effects *in vitro*^46^,^47^. In order to avoid direct immune modulation by trehalose, we worked at a final concentration of 2 mM (0.075% (w/v)). Under these conditions, trehalose does not affect the uptake of beta-ELVs by murine RAW264.7 macrophages as shown by flow cytometry analysis with fluorescently labelled beta-ELVs, and does not increase basal TNF-alpha cytokine secretion. ELISA measurements show consistently higher TNF-alpha stimulation indexes for freeze-thawed beta-ELVs in TRE than in PBS in line with improved conservation of biological activity. While the increased stimulation index relies in part on the increased purity (number of particles per μg of protein) of TRE samples, normalization to particle count shows a greater efficiency of individual particles in TRE.

In conclusion, TRE may allow primary particle populations to be sized more accurately and to reduce extracellular vesicle loss during isolation and storage. Future work on extracellular vesicles obtained from other cell lines and biofluids is required to confirm the potential benefits of TRE. Use of TRE is suitable for a wide range of applications *in vitro* and *in vivo*; TRE can be synthesized chemically, and is compatible with the production of clinical grade exosomes. The ease and cost of TRE make it an appealing alternative to overcome current obstacles linked to the use of frozen extracellular vesicles.

## Materials and methods

### Cell culture

MIN6 cells (kindly provided by Prof. Jun-ichi Miyazaki, University Medical School, Osaka, Japan) were cultured in DMEM high glucose medium (Life Technologies, Saint Aubin, France) supplemented with 10% FCS (Eurobio, Les Ulis, France; cat#CVFSVF00-01; lot#S56441-1267)^24^. Cell cultures were regularly assessed for mycoplasma contamination using the mycoplasma quick test (Euromedex, Souffelweyersheim, France). For exosome production, MIN6 cells were plated at a density of 15 × 10^4^ cells/cm^2^ in DMEM medium. Three days later, the medium was replaced by OptiMEM (Life Technologies; cat#51985-026) supplemented with 1% exosome-precleared FCS obtained through overnight centrifugation at 100,000 × g. Supernatants containing MIN6 exosomes were harvested four days later. At harvest, cell viability was ≥90% in compliance with guidelines to minimize apoptotic body input in exosome preparations^10^,^27^.

RAW264.7 cells (ATCC #TIB-71) were maintained in RPMI 1640 medium (Life Technologies), supplemented with 10% FCS and 2 mM L-glutamine.

### Isolation of beta-ELVs

ELVs were collected from MIN6 supernatants using a method combining differential centrifugation and ultrafiltration steps. Briefly, 200 ml of 96-hours supernatants from confluent MIN6 cells were centrifuged immediately after harvest at 300 × *g* 10 min and filtered through a 0.2 μm filter sterilization device to remove cells, cell debris and microparticles. The remaining supernatant was split and concentrated on an AMICON MWCO-100,000 kDa cellulose ultrafiltration unit (Dutscher, Issy-les-Moulineaux, France; cat#UFC910024) and washed three times with 15 ml of either PBS (Dutscher; cat#21-031-CMR) or PBS 25 mM trehalose (Sigma-Aldrich, Saint Quentin-Fallavier, France; cat#T0167). Approximately, 300 μl of concentrate were recovered, suspended in 10 ml of wash buffer and pelleted twice by 100,000 × g 90 min ultracentrifugation in a SW41 Ti swinging bucket rotor on a L7–55 centrifuge (Beckman Coulter, Villepinte, France) in polyallomer tubes (Beckman Coulter; cat#331372). The final pellet was recovered in approximately 200 μl of PBS or TRE. ELVs were stored at 4 °C for one day or at −80 °C for up to one year. Unless indicated otherwise, experiments were carried out using thawed ELVs.

### Nanoparticle tracking analysis

NTA was performed on a NanoSight LM10 instrument (Malvern Instruments, Orsay, France) with a 488 nm laser and an automated syringe pump at the LNC laboratory (INSERM UMR 866, Dijon, France) using NTA2.3 or NTA3.1 software. After instrument calibration using 100 nm and 200 nm beads, three individual 60 s measurements were recorded for each sample with automated analysis settings for blur, track length and minimum expected particle size.

### Protein analysis

Protein concentration was determined by a modified Bradford protein assay (Fisher Scientific)^8^. Protein size distribution was analysed on an Experion automated electrophoresis system using Pro260 chips (Bio-Rad, Marnes La Coquette, France).

### Immunoblotting

Expression of exosomal markers CD63 and CD81 and cellular calnexin protein was assessed through western blotting. For tetraspanins CD63 and CD81, 15 μg of exosome proteins were separated by non-reducing Laemmli 10% SDS-PAGE and transferred to a PVDF membrane (BioRAD, Marnes la Coquette, France). Membranes were blocked with TBS 4% BSA and then incubated with mAbs NVG-2 1:1000 (CD63) and Eat-2 1:1000 (CD81) purchased from Ozyme (Quentin en Yvelines, France), followed by incubation with HRP-conjugated secondary antibodies 1:20,000 and detection with ECL reagent (Fisher Scientific, Illkirch, France). For blotting with polyclonal rabbit anti-calnexin antibody 1:1000 (Euromedex), proteins were separated under reducing conditions and specific sites were blocked by incubation with TBS 0.1% Tween, 5% milk.

### Cryo-electron tomography

Isolated exosomes were deposited on Formvar carbon-coated, glow-discharged grids. Specimens were prepared using an EM-GP (Leica) under saturated humidity conditions at 20 °C. Mix-Capped gold nanoparticles, 10 nm in diameter^48^, were added to the specimen as fiducial markers. Four μl specimens were deposited on an electron microscope grid (C-flat^TM^), blotted for 0.8 to 1.2 s, and frozen into liquid ethane. Specimen grids were stored in liquid nitrogen until required for observation. Grids were transferred to a single-axis cryo-holder (model 626, Gatan) and were observed using a 200 kV electron microscope (Tecnai G^2^ T20 Sphera, FEI) equipped with a CCD camera (US4000, Gatan). Single-axis tilt series, typically in the angular range ±60°, were acquired under low electron doses (~0.3 e^−^/Å^2^) using the camera in binning mode 2 and at nominal magnifications of 25,000x and 29,000x, corresponding to calibrated pixel sizes of 0.95 and 0.79 nm at the specimen level, respectively. Tomograms were reconstructed using the graphical user interface eTomo from the IMOD software package^49^,^50^. Vesicle dimensions were measured using the slicer in 3dmod. Vesicle axes were measured along the ice slab and perpendicular to the tilt axis.

### Zeta potential

Resistive pulse sensing was performed by Izon on a qNano gold instrument using a 200 nm nanopore and 3.2.2.265 software (Izon Science, Lyon, France).

### Uptake of beta-ELVs by RAW2645.7 macrophages

For beta-ELV internalization assays, 1.7 × 10^8^ MIN6 cells were stained with 5 μM CFDA-SE and maintained in exosome-depleted media. Three days later, beta-ELVs were harvested and used to treat 1 × 10^5^ RAW264.7 cells cultured in suspension for 6 hours at 37 °C. Untreated cells and beta-ELV-treated cells cultured at 4 °C were included as negative controls. Cellular uptake was analysed by flow cytometry gating on single 7-AAD -negative living cells on a FACSAria instrument (BD Biosciences, Le Pont de Claix, France). Results were analysed using FlowJo v10.0.8 Treestar software (Miltenyi BioTEC, Paris, France).

### TNF-alpha cytokine secretion assays

RAW cells were plated at 50 × 10^3^ cells/well, in 96-well plates, 5 hours before exosome treatment followed by 18 h of treatment with unlabelled exosomes. The final concentration of trehalose was adjusted to 2 mM for all doses of TRE beta-ELVs. Cytokine content of culture supernatants was measured using mouse TNF-alpha ELISA kits (Ozyme).

### Statistical analysis

Statistical tests were performed using Prism GraphPad Software (Comparex, Issy-les-Moulineaux, France) using tests as indicated in the figure legends.

## Additional Information

**How to cite this article**: Bosch, S. *et al*. Trehalose prevents aggregation of exosomes and cryodamage. *Sci. Rep.* **6**, 36162; doi: 10.1038/srep36162 (2016).

**Publisher’s note:** Springer Nature remains neutral with regard to jurisdictional claims in published maps and institutional affiliations.

## Supplementary Material

Supplementary Information

## References

[b1] Harding C., Heuser J. & Stahl P. Receptor-mediated endocytosis of transferrin and recycling of the transferrin receptor in rat reticulocytes. Journal of Cell Biology 97, 329–339, doi: 10.1083/jcb.97.2.329 (1983). 6309857 PMC2112509

[b2] Pan B. T. & Johnstone R. M. Fate of the transferrin receptor during maturation of sheep reticulocytes in vitro-selective externalization of the receptor. Cell 33, 967–977, doi: 10.1016/0092-8674(83)90040-5 (1983). 6307529

[b3] Raposo G. . B lymphocytes secrete antigen-presenting vesicles. J Exp Med. 183, 1161–1172 (1996). 8642258 10.1084/jem.183.3.1161 PMC2192324

[b4] Ramachandra L. . Mycobacterium tuberculosis Synergizes with ATP To Induce Release of Microvesicles and Exosomes Containing Major Histocompatibility Complex Class II Molecules Capable of Antigen Presentation. Infection and Immunity 78, 5116–5125, doi: 10.1128/iai.01089-09 (2010). 20837713 PMC2981298

[b5] Skog J. . Glioblastoma microvesicles transport RNA and proteins that promote tumour growth and provide diagnostic biomarkers. Nature Cell Biology 10, 1470–U1209, doi: 10.1038/ncb1800 (2008). 19011622 PMC3423894

[b6] Yanez-Mo M. . Biological properties of extracellular vesicles and their physiological functions. Journal of Extracellular Vesicles 4, doi: 10.3402/jev.v4.27066 (2015). PMC4433489 25979354

[b7] Salama A. . MicroRNA-29b Modulates Innate and Antigen-Specific Immune Responses in Mouse Models of Autoimmunity. Plos One 9, doi: 10.1371/journal.pone.0106153 (2014). PMC4159199 25203514

[b8] Théry C., Amigorena S., Raposo G. & Clayton A. Isolation and characterization of exosomes from cell culture supernatants and biological fluids. Curr Protoc Cell Biol. Chapter 3, Unit 3.22, doi: 10.1002/0471143030.cb0322s30 (2006). 18228490

[b9] Lasser C., Eldh M. & Lotvall J. Isolation and characterization of RNA-containing exosomes. Journal of visualized experiments: JoVE e3037, doi: 10.3791/3037 (2012). PMC3369768 22257828

[b10] Witwer K. W. . Standardization of sample collection, isolation and analysis methods in extracellular vesicle research. Journal of extracellular vesicles 2, doi: 10.3402/jev.v2i0.20360 (2013). PMC3760646 24009894

[b11] Jain N. K. & Roy I. Effect of trehalose on protein structure. Protein Science 18, 24–36, doi: 10.1002/pro.3 (2009). 19177348 PMC2708026

[b12] Eroglu A., Toner M. & Toth T. L. Beneficial effect of microinjected trehalose on the cryosurvival of human oocytes. Fertility and Sterility 77, 152–158, doi: 10.1016/s0015-0282(01)02959-4 (2002). 11779606

[b13] Katenz E. . Cryopreservation of primary human hepatocytes: The benefit of trehalose as an additional cryoprotective agent. Liver Transplantation 13, 38–45, doi: 10.1002/lt.20921 (2007). 17154395

[b14] Ikeda M. . Clinical application of ET-Kyoto solution for lung transplantation. Surgery Today 45, 439–443, doi: 10.1007/s00595-014-0918-0 (2015). 24845738

[b15] Beattie G. M. . Trehalose: A cryoprotectant that enhances recovery and preserves function of human pancreatic islets after long-term storage. Diabetes 46, 519–523, doi: 10.2337/diabetes.46.3.519 (1997). 9032112

[b16] Kim Y.-C. . Influenza immunization with trehalose-stabilized virus-like particle vaccine using microneedles. 3rd Vaccine Global Congress, Singapore 2009 2, 17–21, doi: 10.1016/j.provac.2010.03.004 (2010). PMC3082143 21528098

[b17] Richards A. B. . Trehalose: a review of properties, history of use and human tolerance, and results of multiple safety studies. Food and Chemical Toxicology 40, 871–898, doi: 10.1016/s0278-6915(02)00011-x (2002). 12065209

[b18] Sato S., Okamoto K., Minami R., Kohri H. & Yamamoto S. Trehalose can be used as a parenteral saccharide source in rabbits. Journal of Nutrition 129, 158–164 (1999). 9915893 10.1093/jn/129.1.158

[b19] Beranger F., Crozet C., Goldsborough A. & Lehmann S. Trehalose impairs aggregation of PrPSc molecules and protects prion-infected cells against oxidative damage. Biochemical and Biophysical Research Communications 374, 44–48, doi: 10.1016/j.bbrc.2008.06.094 (2008). 18602368

[b20] Liu R., Barkhordarian H., Emadi S., Park C. B. & Sierks M. R. Trehalose differentially inhibits aggregation and neurotoxicity of beta-amyloid 40 and 42. Neurobiology of Disease 20, 74–81, doi: 10.1016/j.nbd.2005.02.003 (2005). 16137568

[b21] Tanaka M. . Trehalose alleviates polyglutamine-mediated pathology in a mouse model of Huntington disease. Nature Medicine 10, 148–154, doi: 10.1038/nm985 (2004). 14730359

[b22] Maki K. C., Kanter M., Rains T. M., Hess S. P. & Geohas J. Acute effects of low insulinemic sweeteners on postprandial insulin and glucose concentrations in obese men. International Journal of Food Sciences and Nutrition 60, 48–55, doi: 10.1080/09637480802646923 (2009). 19180358

[b23] Hood J. L., Scott M. J. & Wickline S. A. Maximizing exosome colloidal stability following electroporation. Analytical Biochemistry 448, 41–49, doi: 10.1016/j.ab.2013.12.001 (2014). 24333249 PMC3954633

[b24] Miyazaki J. I. . Establishment of a pancreatic beta-cell line that retains glucose-inducible insulin-secretion - special reference to expression of glucose transporter isoforms. Endocrinology 127 (1990). 10.1210/endo-127-1-126 2163307

[b25] Gardiner C., Ferreira Y. J., Dragovic R. A., Redman C. W. G. & Sargent I. L. Extracellular vesicle sizing and enumeration by nanoparticle tracking analysis. Journal of extracellular vesicles 2, doi: 10.3402/jev.v2i0.19671 (2013). PMC3760643 24009893

[b26] Gyoergy B. . Membrane vesicles, current state-of-the-art: emerging role of extracellular vesicles. Cellular and Molecular Life Sciences 68, 2667–2688, doi: 10.1007/s00018-011-0689-3 (2011). 21560073 PMC3142546

[b27] Van Deun J. . The impact of disparate isolation methods for extracellular vesicles on downstream RNA profiling. Journal of extracellular vesicles 3, doi: 10.3402/jev.v3.24858 (2014). PMC4169610 25317274

[b28] Sheng H. . Insulinoma-Released Exosomes or Microparticles Are Immunostimulatory and Can Activate Autoreactive T Cells Spontaneously Developed in Nonobese Diabetic Mice. Journal of Immunology 187, 1591–1600, doi: 10.4049/jimmunol.1100231 (2011). PMC3150365 21734072

[b29] Kim S. H. . Exosomes derived from IL-10-treated dendritic cells can suppress inflammation and collagen-induced arthritis. Journal of Immunology 174, 6440–6448 (2005). 10.4049/jimmunol.174.10.6440 15879146

[b30] Bashratyan R., Sheng H., Regn D., Rahman M. J. & Dai Y. D. Insulinoma-released exosomes activate autoreactive marginal zone-like B cells that expand endogenously in prediabetic NOD mice. European Journal of Immunology 43, 2588–2597, doi: 10.1002/eji.201343376 (2013). 23817982 PMC3832688

[b31] Zhou H. . Collection, storage, preservation, and normalization of human urinary exosomes for biomarker discovery. Kidney International 69, 1471–1476, doi: 10.1038/sj.ki.5000273 (2006). 16501490 PMC2276656

[b32] Linares R., Tan S., Gounou C., Arraud N. & Brisson A. R. High-speed centrifugation induces aggregation of extracellular vesicles. Journal of Extracellular Vesicles 4, doi: 10.3402/jev.v4.29509 (2015). PMC4689953 26700615

[b33] Kirkegaard J. S. . Xenotropic retrovirus Bxv1 in human pancreatic beta cell lines. Journal of Clinical Investigation 126, 1109–1113, doi: 10.1172/jci83573 (2016). 26901817 PMC4767346

[b34] Willms E. . Cells release subpopulations of exosomes with distinct molecular and biological properties. Scientific Reports 6, doi: 10.1038/srep22519 (2016). PMC4773763 26931825

[b35] Bobrie A., Colombo M., Krumreich S., Raposo G. & Théry C. Diverse subpopulations of vesicles secreted by different intracellular mechanisms are present in exosome preparations obtained by differential ultracentrifugation. Journal of Extracellular Vesicles 1, doi: 10.3402/jev.v1i0.18397 (2012). PMC3760636 24009879

[b36] Hoog J. L. & Lotvall J. Diversity of extracellular vesicles in human ejaculates revealed by cryo-electron microscopy. Journal of Extracellular Vesicles 4, doi: 10.3402/jev.v4.28680 (2015). PMC4643196 26563734

[b37] Arraud N. . Extracellular vesicles from blood plasma: determination of their morphology, size, phenotype and concentration. Journal of Thrombosis and Haemostasis 12, 614–627, doi: 10.1111/jth.12554 (2014). 24618123

[b38] Webber J. & Clayton A. How pure are your vesicles? Journal of extracellular vesicles 2, doi: 10.3402/jev.v2i0.19861 (2013). PMC3760653 24009896

[b39] Marimpietri D. . Proteome Profiling of Neuroblastoma-Derived Exosomes Reveal the Expression of Proteins Potentially Involved in Tumor Progression. Plos One 8, doi: 10.1371/journal.pone.0075054 (2013). PMC3777909 24069378

[b40] Smith Z. J. . Single exosome study reveals subpopulations distributed among cell lines with variability related to membrane content. Journal of Extracellular Vesicles 4, doi: 10.3402/jev.v4.28533 (2015). PMC4673914 26649679

[b41] Yang L. . Exogenous trehalose largely alleviates ionic unbalance, ROS burst, and PCD occurrence induced by high salinity in Arabidopsis seedlings. Frontiers in Plant Science 5, doi: 10.3389/fpls.2014.00570 (2014). PMC4212613 25400644

[b42] Chang B., Yang L., Cong W., Zu Y. & Tang Z. The improved resistance to high salinity induced by trehalose is associated with ionic regulation and osmotic adjustment in Catharanthus roseus. Plant Physiology and Biochemistry 77, 140–148, doi: 10.1016/j.plaphy.2014.02.001 (2014). 24589477

[b43] Reina-Bueno M. . Role of Trehalose in Salinity and Temperature Tolerance in the Model Halophilic Bacterium Chromohalobacter salexigens. Plos One 7, doi: 10.1371/journal.pone.0033587 (2012). PMC3308980 22448254

[b44] Sokolova V. . Characterisation of exosomes derived from human cells by nanoparticle tracking analysis and scanning electron microscopy. Colloids and Surfaces B-Biointerfaces 87, 146–150, doi: 10.1016/j.colsurfb.2011.05.013 (2011). 21640565

[b45] Jayachandran M., Miller V. M., Heit J. A. & Owen W. G. Methodology for isolation, identification and characterization of microvesicles in peripheral blood. Journal of Immunological Methods 375, 207–214, doi: 10.1016/j.jim.2011.10.012 (2012). 22075275 PMC3253871

[b46] Tanaka K. . Trehalose does not affect the functions of human neutrophils *in vitro*. Surgery Today 44, 332–339, doi: 10.1007/s00595-013-0625-2 (2014). 23700245

[b47] Echigo R. . Trehalose treatment suppresses inflammation, oxidative stress, and vasospasm induced by experimental subarachnoid hemorrhage. Journal of Translational Medicine 10, doi: 10.1186/1479-5876-10-80 (2012). PMC3422174 22546323

[b48] Duchesne L., Gentili D., Comes-Franchini M. & Fernig D. G. Robust Ligand Shells for Biological Applications of Gold Nanoparticles. Langmuir 24, 13572–13580, doi: 10.1021/la802876u (2008). 18991409

[b49] Mastronarde D. N. Dual-axis tomography: An approach with alignment methods that preserve resolution. Journal of Structural Biology 120, 343–352, doi: 10.1006/jsbi.1997.3919 (1997). 9441937

[b50] Kremer J. R., Mastronarde D. N. & McIntosh J. R. Computer visualization of three-dimensional image data using IMOD. Journal of Structural Biology 116, 71–76, doi: 10.1006/jsbi.1996.0013 (1996). 8742726

