# Supplementary material for: Trehalose prevents aggregation of exosomes and cryodamage
Source: Sci Rep. 2016 Nov 8;6:36162. doi: 10.1038/srep36162 (PMC5099918; doi:10.1038/srep36162)
Supplement: Supplementary Information [file srep36162-s1.pdf]

Trehalose prevents aggregation of exosomes and cryodamage

Steffi Bosch[1], Laurence de Beaurepaire[1], Marie Allard[1], Mathilde Mosser[1], Claire Heichette[2], Denis Chretien[2,3], Dominique Jegou[1], Jean-Marie Bach[1]

[1] IECM, EA4644 Nantes University, ONIRIS, USC1383 INRA, Nantes, France.

[2] IGDR, UMR6290 CNRS, University of Rennes 1, Rennes France

[3] MRIC-Biosit, UMS3480 CNRS, University of Rennes 1, Rennes France

Address for correspondence: Steffi Bosch, IECM, Oniris, Atlanpole, La Chantrerie, CS40706, F-44307 Nantes cedex 3, France; Tel.: +33 (0) 240 687 831; Fax: +33 (0)240 687 805; Email: steffi.bosch@oniris-nantes.fr

Supplementary Information

Supplementary Materials and methods

OptiPrep™ density gradient centrifugation

A discontinuous 5-40% iodixanol gradient was prepared using OptiPrep™ (Sigma-Aldrich; cat#D1556) and conditions described by [27]. 100µg in 600µl of ELVs were overlaid onto the gradient and centrifuged for 18h at 100,000 x *g* 4° C. Fractions of 1 ml were collected and their refractive index was determined using an Atago Master-Sur/NM refractometer (Dutscher). Fraction density was calculated based on the supplier's conversion of refractive indexes (http://www.axis-shield-density-gradient-media.com/Preparation%20of%20gradient%20solutions.pdf). Fractions were washed in PBS or TRE and centrifuged for 3h at 100,000 x *g* 4° C. The final pellet was suspended in 50 µl of PBS or TRE and subjected to protein analysis.

Supplementary Table

Supplementary Table S1. PSD of beta-ELVs from NTA measurements

	D50 (nm)	Span $= \frac{D90-D10}{D50}$	Pearson's skewness coefficient $= 3x \frac{(Mean-mode)}{SD}$	Mode (nm)	Mean (nm)	SD (nm)	Nb part.(x E7)/ml Culture supernatant	Nb part.(xE8)/ µg of protein	Zeta Potential (mV)
PBS	136.5 (126.0-153.0)	1.4 (1.1-1.9)	1.5 (1.1-4.3)	113 (46-130)	158 (140-179)	88 (60-105)	7.2 (1.6-40.5)	2.2 (0.4-13.3)	-20.7 (-24.7- (-15.4))
TRE	123.5 (115.0-135.0)	1.2 (1.0-1.4)	1.3 (0.9-1.8)	111 (102-122)	139 (126-154)	67 (55-80)	24.5 (8.1-61.9)	6.7 (0.8-19.6)	-19.6 (-33.2- (-17.3))
P value	0.0015	0.0234	0.0405	0.0566	0.0005	0.0020	0.0005	0.0010	0.3125

Median (range) values obtained from n=12 samples, p-values calculated using a two-tailed Wilcoxon matched-pairs signed rank test.

Supplementary Figures

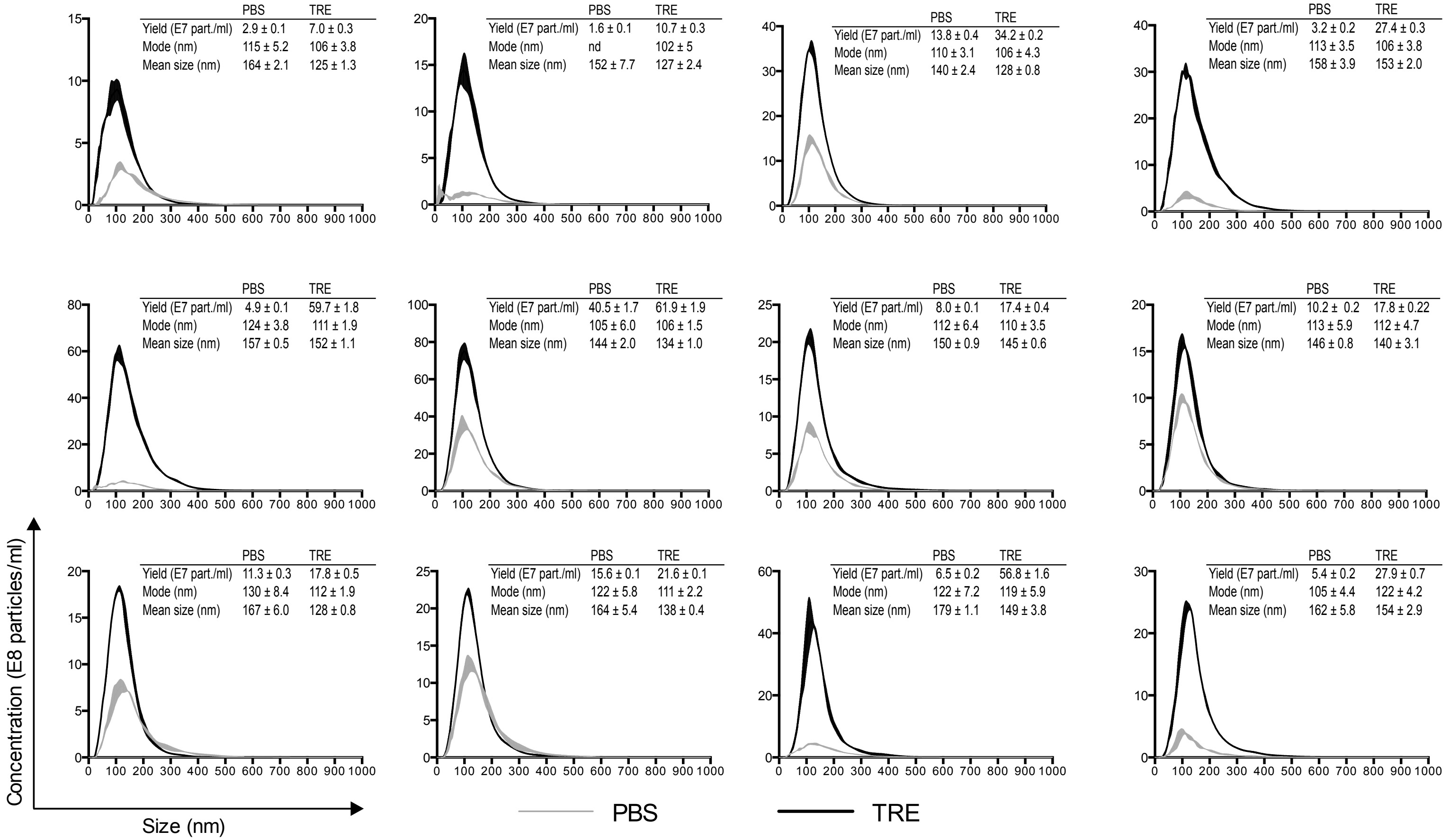

Supplementary Figure S1. Particle size distribution of MIN6 ELVs in PBS or TRE *analysed using Nanoparticle Tracking Analysis. Overlays of mean particle size distribution ± standard error (shaded area) of EVs in PBS (grey line) or TRE (black line) were prepared using GraphPad. Particle yield per millilitre of culture supernatant, mean and mode size are reported for each of the twelve independent productions.*

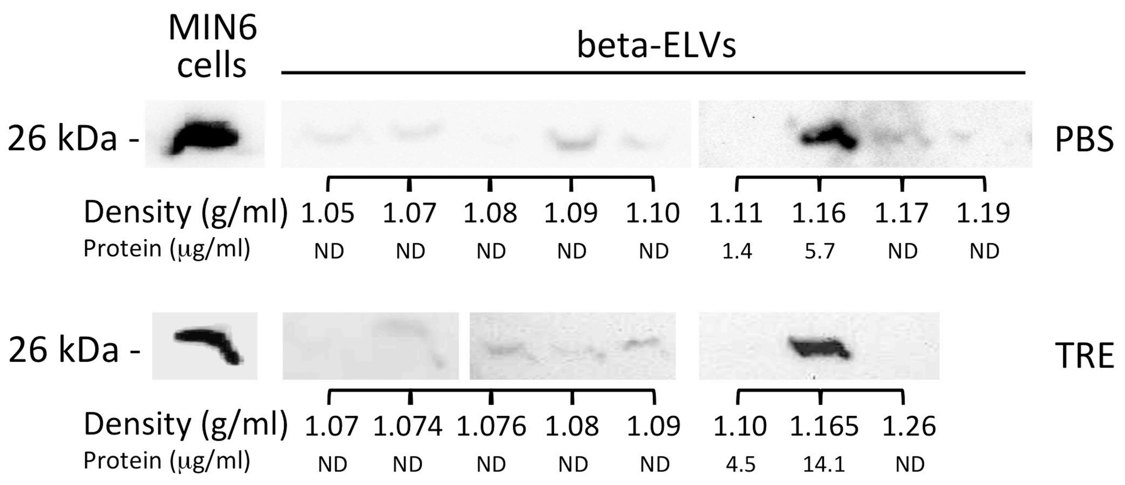

Supplementary Figure S2. Particle size distribution of MIN6 ELVs in PBS or TRE *analysed using Nanoparticle Tracking Analysis. Overlays of mean particle size distribution ± standard error (shaded area) of EVs in PBS (grey line) or TRE (black line) were prepared using GraphPad. Particle yield per millilitre of culture supernatant, mean and mode size are reported for each of the twelve independent productions.*

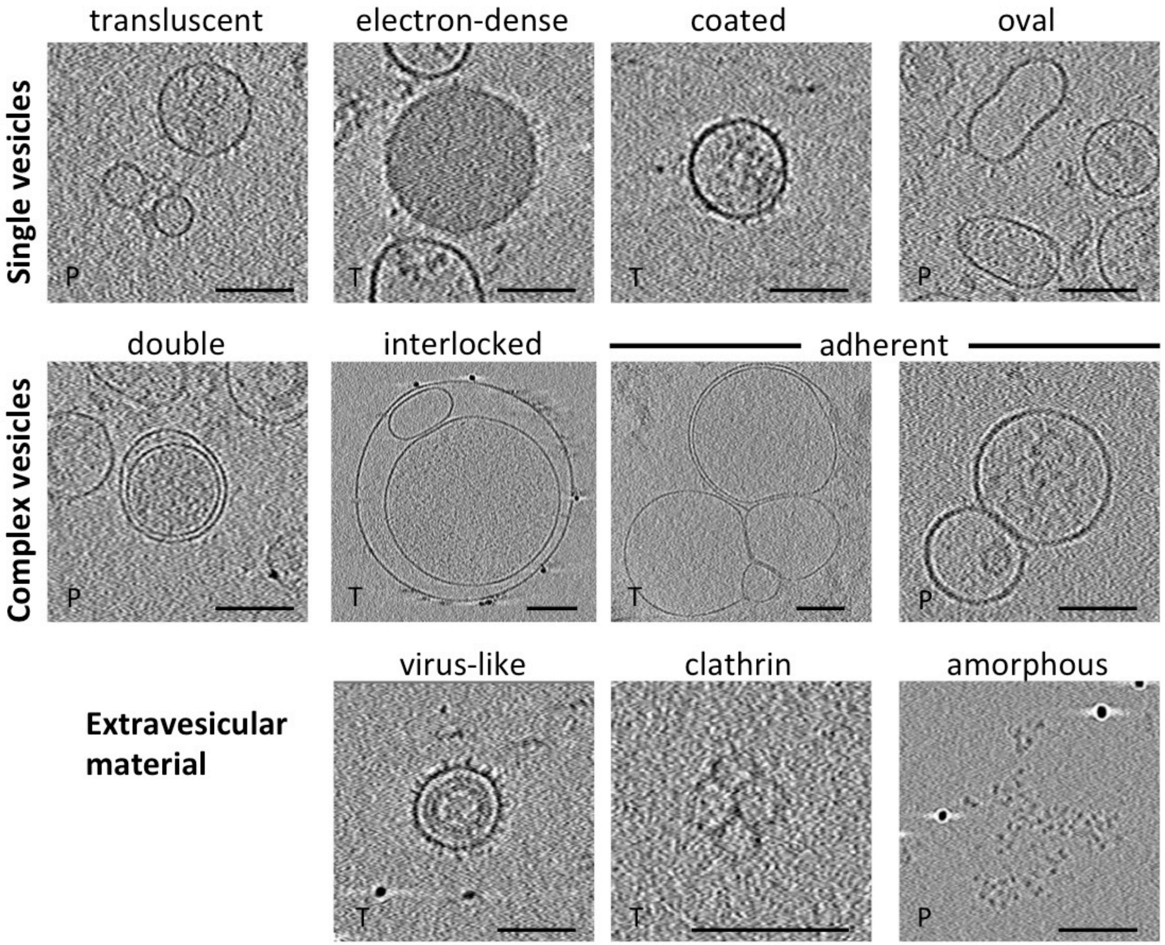

Supplementary Figure S3. *Cryo-electron tomography analysis revealed similar pleiomorphic subpopulations of beta-ELVs in PBS and TRE (indicated by the letter P or T, respectively) delineated by double membrane bilayers. Single vesicles differed in size, shape, electron density and presence of surface proteins. Single translucent vesicles were the prevailing subcategory. No incomplete vesicles indicative of lysis were observed. Multivesicular structures were composed of double, interlocked or adherent vesicles. Extravesicular material included virus-like particles, clathrin triskelion-like structures and amorphous material. Images were acquired at a magnification of x 25,000 or 29,000. Scale bars = 100 nm.*
